# Prevalence and risk factors of human brucellosis and malaria among patients with fever in malaria-endemic areas, attending health institutes in Awra and Gulina district, Afar Region, Ethiopia

**DOI:** 10.1186/s12879-021-06654-y

**Published:** 2021-09-10

**Authors:** Sintayehu Mehari, Biruk Zerfu, Kassu Desta

**Affiliations:** 1 Federal Police Hospital, Addis Ababa, Ethiopia; 2 Department of Medical Laboratory Sciences, College of Health Sciences, Addis Ababa University, P.O. Box 1176, Addis Ababa, Ethiopia

**Keywords:** Brucellosis, Malaria, Prevalence, Febrile, Risk factors, Ethiopia

## Abstract

**Background:**

Brucellosis is an important neglected bacterial zoonotic disease that has been affecting animals and humans for decades. Malaria has been considered major cause of illness in tropical areas, including Ethiopia. This study aimed to identify prevalence and risk factors of human brucellosis and malaria among patients with fever in malaria-endemic areas attending health institutes in Awra and Gulina district, Afar Region, Ethiopia.

**Methods:**

A purposive cross-sectional study was conducted among febrile patients who attended health institutes in Awra and Gulina district of Afar region from February to May 2019. 3–5 ml blood samples were collected, thick and thin blood films were prepared and examined for malaria; serum was separated and tested for anti-*Brucella* using Rose Bengal Plate Test, and the seropositives were subjected to ELISA. Data were entered using EpiData3.1 and analyses were performed using Stata SE 14.

**Results:**

A total of 444 febrile individuals (59.5% female) of age ranging from 2 to 83 years (mean = 26.1, SD =  ± 11.8) were participated in this study. The overall seroprevalence of brucellosis was 31.5% (95% CI; 27.4–36.0%) by RBPT and 15.8% (95% CI; 12.7–19.7%) by ELISA, as well as the prevalence of malaria (*P. falciparum*) was 4.3% (95% CI; 2.7–6.6%) among febrile patients. Malaria was more common in males (7.2% 95% CI; 4.2–12.1%) than in female (2.3% 95% CI; 1.0–5.0%, p = 0.01) and in non-married than in married (7.6% 95% CI; 4.1–13.6% vs. 2.9% 95% CI; 1.5–5.4%, p = 0.02). Being male (AOR = 2.41, 95%CI: 1.36**–**4.26, p < 0.002), drinking raw milk (AOR = 26.68, 95%CI: 3.22- 221.13, p = 0.002) and boiled milk (AOR = 17.52, 95%CI: 2.06—149.04, p = 0.009**)** and touching aborted fetus/discharges without protective (AOR = 2.56, 95%CI: 1.01–6.528.50, p = 0.048) were independently associated with brucellosis among febrile patients.

**Conclusion:**

The prevalence of brucellosis in fever patients in this study area is higher than malaria. Consumption of raw milk and contact with animal discharge can cause significant risk of *Brucella* infection. So, brucellosis disease must be sought in the differential diagnosis, like ELISA test that can be used to differentiate from other febrile diseases like malaria.

## Background

Brucellosis is a neglected bacterial zoonotic disease that has been affecting animals and humans for years [1]. The annual global human brucellosis case reports are about half a million [2]. The poor surveillance systems in developing countries like Ethiopia have led to the underestimation of the true burden of the cases of brucellosis [3]. Several developed countries eradicated brucellosis, but it remains endemic in northern and eastern Africa, India, Central Asia, Mexico, and central and southern America [4]. In Sub-Saharan Africa, animal brucellosis ranges from 10.2 to 25.7% [5]. This high distribution in animals makes human beings to be exposed to acquire the infection and have a potential threat of re-emergence in several countries with an increased incidence of infection in cattle [5].

Brucellosis is an occupational hazard for veterinarians, laboratory workers, slaughterhouse workers, and farmers which can be acquired through either contact with infected animals, their tissues, or animal products. The bacteria enter humans through wounds or abrasion of skins/mucous membranes during contact with infected animals and during consumption of raw or unpasteurized milk and dairy products of such milk [6]. Brucellosis in humans is manifested mainly by intermittent or irregular fever, headache, weakness, profuse sweating, chills, arthralgia, depression, weight loss, hepatomegaly and splenomegaly[7] and rarely arthritis, spondylitis, osteomyelitis, epididymitis, and orchitis, but in severe cases, neuro brucellosis, liver abscesses, and endocarditis have been reported [8].

Since human brucellosis has wide clinical feature presentations, it mimics many communicable and non-communicable diseases like malaria, typhoid fever, typhus, rheumatic fever, joint diseases, and others. These features pose a diagnostic difficulty for brucellosis, especially in developing countries like Ethiopia, because they adhere mostly to apparent clinical signs and symptoms, as diagnostic indicators to rule out diseases. In Ethiopia, the determination of risk factors and health intervention of human brucellosis is not routinely undertaken due to the lack of effective and appropriate diagnostic facilities [9, 10].

On the other hand, 75% of Ethiopia’s landmass is favorable for malaria transmission, leaving about 68% of the total population at risk of malaria [11]. However, Ethiopia scaled-up malaria intervention programs towards elimination that have achieved a 40% reduction of malaria cases and increased capacity of case confirmation of presumed malaria diagnosis from 54% in 2013 to 87% in 2017 [12]. However, certain infections with clinical symptoms comparable to malaria, such as brucellosis, have been left undetected and untreated as a result of the intervention. The current study area is pastoral and agro-pastoral that rears camel, sheep, goat, and cattle. Some studies have shown that animal brucellosis is highly distributed and the livelihood of the population is very close to animals that create potential risk factors to acquire brucellosis [13–15]. Nevertheless, human brucellosis has rarely been surveyed either as misdiagnosed or abandoned at all due to similarity of signs and symptoms presumably with malaria or unfamiliarity of health care workers with the disease and its epidemiology in this area [16, 17]. The aim of this study was to determine the prevalence of human brucellosis and malaria among patients with fever in malaria-endemic areas, attending health institutes in Awra and Gulina district, Afar Region, Ethiopia. In addition, the study aimed to identify potential risk factors of human brucellosis among febrile patients of the indicated study area.

## Methods

### Study setting and population

The study was conducted in Kelwani primary hospital and Derayitu health center of Awra and Gulina district of Afar Region, Northeast of Ethiopia. The majority of the communities are pastoralists whose livelihoods depend on livestock, specifically camels, cattle, and small ruminants while few are practicing agro-pastoralism and growing crops by irrigation of Awash River.

### Study design and sample size determination

A health-institution-based cross-sectional study design was used to identify prevalence and risk factors of human brucellosis and malaria among patients with fever in malaria-endemic areas, attending health institutes in Awra and Gulina district of Afar region, Ethiopia, from February to May 2019. The finding of previous community-based seroprevalence of brucellosis (4.4%) in other pastoral areas of the region’s community was used to estimate the sample size [17]. Based on this information, the calculated sample size, at 95% confidence level, 5% degree of accuracy, and with 10% compensation for refusal, was 444 respondents.

### Study participants, sample and data collection

All patients older than two years who had any duration of fever and measured axial body temperature ≥ 37.5 °C during data collection period, and were willing to provide written consent/assent for participation was recruited to the study. A total of 444 respondents were interviewed in their local language (Afar language) using a structured questionnaire to collect sociodemographic characteristics, including gender, age, educational status, marital status, occupational status, residence (urban/rural), and potential risk factors like the type of animal owned and milk sources (large ruminants, small ruminates or camels), ways of milk consumption either raw or boiled, the experience of milk consumption from aborted animals, exposure to aborted fetus/ materials of the animal without protective equipment, and the clinical symptoms they felt and the duration of the symptoms. A 3–5 ml of venous blood was collected from each febrile patient using a plain vacutainer tube. Thin and thick blood smears were prepared immediately from each blood sample for the diagnosis of malaria. The remaining sample was kept at room temperature for 30 min to facilitate clotting and centrifuged at 3000 rpm for 5 min to get the clear serum. All sera were separated in a labeled 1.8 ml Cryotubes, transported to Addis Ababa Federal police laboratory in a cold box, and stored at 4 °C until testing.

### Blood examination for malaria

Malaria was detected from Giemsa stained blood films following the guideline of the Ethiopian Ministry of Health for the diagnosis of malaria and identification of *Plasmodium* species at the health institute [18].

### Blood examination for brucellosis

Two types of serological tests were used to determine the seroprevalence of brucellosis.

The sera were screened using Rose Bengal Plate Test (RBPT) and positive reactors were further subjected to ELISA. All sera and RBPT reagent and controls were taken out from the refrigerator and kept at room temperature for 30 min to screen for anti-*Brucella* antibodies in the Addis Ababa Federal police laboratory. As previously described [19], the smooth, attenuated stained *Brucella* antigen suspension was mixed with positive and negative controls and serum on a circular test card. If a specific antibody to *Brucella* antigen is present in the serum, it reacts with the antigen suspension to produce visible agglutination after shaking on a low-speed shaker for four minutes. No agglutination indicates an absence of specific antibodies to *Brucella* antigens. All sera positive for *Brucella* antibody using RBPT were transported to Armeaur Hansen Research Institute (AHRI) to confirm the anti-*Brucella* antibodies by IgG ELISA. According to the manufacturer’s guideline (Demeditec *Brucella abortus* IgG ELISA DEBRU01, Germany), qualitative anti-*Brucella* IgG ELISA was determined based on the principle of the spectrophotometric enzyme immunoassay at the wavelength of 450 nm. The calculated absorption for the patients’ sera was compared with the value of the cut-off standard, 10 IU/ml [20]. If the value of the sample was higher than the cut-off standard, it was considered as positive whereas below the cut-off standard, the result was considered negative.

### Data analysis

Descriptive analysis was used to summarize the data in the form of frequencies and percentages. Pearson Chi2 test was used for testing relationships between brucellosis and malaria infection with each demographic characteristic of study participants. Univariate logistic regression analyses were conducted to establish the association of the putative risk factors with brucellosis and odds ratio at 95% confidence intervals (CI) was considered. All risk factors significant at univariate analysis were considered for multivariate logistic regression analysis to determine the independent association between risk factors and brucellosis at 95% CI. A P-value below 0.05 was considered statistical significance.

### Ethical Consideration

This study received ethical clearance from the Ethical Review Board of the Department of Medical Laboratory Science, College of Health Sciences, Addis Ababa University (DRERC/410/19/MLS). Permission was obtained from Derayitu Health center and Kelwani Primary Hospital. Participants’ information sheet, which contains the objective of the study, inclusion/exclusion criteria, the required data and methods of data collection as well as informed consent/assent document, was prepared in the Amharic language, the national language of the country. Then, the elements of the participants’ information sheet initially were orally translated to the local language (Afar Language) and described to each of the study participants or parents in the case of children under 18 years by trained local health personnel. Informed consent/assent was obtained from each participant and/or parent for children aged between 12 and 18 years. A blood sample was collected under aseptic conditions by experienced laboratory technicians. Study participants who were found positive for malaria were treated according to malaria treatment guidelines and the rest were treated with different antibiotics accordingly as per clinician presumptive diagnosis.

## Results

### Sociodemographic characteristics

A total of 444 febrile individuals (female, 59.5%), with the age range of 2 to 83 (mean = 26.1, SD =  ± 11.8) years participated in the study, with the majority, 241 (54.3%) of the participants between 15–29 years old. Among 444 febrile study participants, 249 (56.1%) were agro- and/or pastoralists 201 (45.3%) were illiterate, 313 (70.5%) married, and 347 (78.1%) were rural residents (Table 1). The clinical symptoms were fever 444 (100%), headache 340 (76.6%), vomiting 139 (31.1%), general malaise 128 (28.6%), joint pain 125 (28.2%) and general weakness 118 (26.6%) and the duration of the reported illness ranged from 1 to 30 days, with most of the patients 289 (65.1%) felt the illness for the duration of 1–3 days.

**Table 1:** Demographic characteristics and distribution of brucellosis among study respondents (N = 444)

Factors	Tested N (%)	RBPT^+ve^ N (%)	ELISA^+ve^ N (%)	Total + ve N (%; 95% CI)	P value
Gender					
Male	180 (40.5)	68 (37.8)	42 (61.8)	42 (23.3;17.7–30.1)	** < 0.001**
Female	264 (59.5)	72 (27.3)	28 (38.9)	28 (10.6;7.4–15.0)	
Age					
2–14	58 (13.1)	18 (31.0)	7 (38.9)	7 (12.1;5.8–23.4)	0.311
15–29	241 (54.3)	77 (32.0)	37 (48.1)	37 (15.4;12.2–20.5)	
30–44	111 (25.0)	34 (30.6)	17 (50.0)	17 (15.3;5.4–24.4)	
≥ 45	34 (7.6)	11 (32.4)	9 (81.8)	9 (26.5;14.2–43.9)	
Education					
Illiterate	201 (45.3)	71 (35.3)	41 (57.8)	41 (20.4;15.4–26.6)	**0.045**
Primary school and above	243 (54.7)	69 (28.4)	29 (40.0)	29 (11.9;8.4–16.7)	
Marital status					
Married	313 (70.5)	99 (31.9)	52 (52.3)	52 (16.6;12.9–21.2)	0.449
Non married	131 (29.5)	41 (31.3)	18 (43.9)	18 (13.7;8.8–20.8)	
Residence					
Urban	97 (21.9)	23 (23.7)	9 (39.1)	9 (9.3;4.9–17.0)	**0.047**
Rural	347 (78.1)	117 (33.7)	61 (52.1)	61 (17.6;13.9–22.0)	
Occupation					
Pastoralist	215	77 (35.8)	43 (55.8)	43 (20.0;15.2–25.9)	**0.018**
Others^a^	229	63 (27.5)	27 (42.9)	27 (11.8;8.2–16.7)	
Type of animal owned					
None	111	20 (18.0)	8 (40.0)	8 (7.2;3.6–13.8)	**; 0.032**
Large ruminant	25	15 (60.0)	7 (46.7)	7 (28.0;13.7–48.7)	
Small ruminant	62	28 (45.2)	13 (46.4)	13 (21.0;12.5–33.0)	
Camel	26	5 (19.3)	4 (80.0)	4 (15.4;5.8–35.1)	
Two or more types of animals	220	72 (32.7)	38 (52.8)	38 (17.3;12.8–22.9)	
Malaria					
Negative	425	130 (30.6)	65 (50.0)	65 (15.3;12.2–19.1)	0.197
Positive	19	10 (52.6)	5 (50.0)	5 (26.3;11.1–50.1)	

^a^Agro-pastoralist, Daily laborer, Governmental workers and students

### Laboratory results

Of all 444 tested sera for brucellosis, the seroprevalence of brucellosis was found 140 (31.5%) by RBPT. From all seropositive, 70 (50%) were found positive by ELISA. The combined seroprevalence was found 70/444 (15.8%; 95% CI; 12.7–19.7%). Brucellosis was frequently detected in males than in females (23.3%; 95% CI; 17.7–30.1% vs. 10.6%; 95% CI; 7.4–15.0%, p < 0.001), in illiterate than in primary school and above educational status (20.4%; 95% CI; 15.4–26.6 vs. 11.9%; 95%CI; 8.4–16.7% p = 0.045) and in rural residents than in urban residents (17.6%; 95% CI; 13.9–22.0% vs. 9.3%; 95% CI; 4.9–17.0%p = 0.041). The brucellosis disease was more frequently detected among pastoralists than among a group that holds agro-pastoralist, daily laborers, governmental workers, and students (20.0%; 95% CI; 15.2–25.9% vs. 11.8%; 95% CI; 8.2–16.7%, p = 0.018). Similarly, large ruminant owners had the highest rate of brucellosis, followed by small ruminant owners, more than one animal type owners, and camel owners when compared to those who do not have any animals (28%, 21%, 17.3%, 15.4% vs. 7.2, p = 0.032). On the other hand, brucellosis was more frequently detected among malaria positive (26.3%; 95% CI; 11.1–50.1%) than among malaria negative (15.3%; 95% CI; 12.2–19.1%) but the difference was not statistically significant (p = 0.197) (Table 1).

Among all febrile patients (444) tested for malaria, 19 (4.3%, 95% CI; 2.7–6.6%) were found positive for malaria (*P. falciparum*) by microscopic detection of Giemsa stained thick and thin blood films. Malaria cases were more common among males than females (7.2% 95% CI; 4.2–12.1% vs. 2.3% 95% CI; 1.0–5.0%, p = 0.01) and non-married than married (7.6% 95% CI; 4.1–13.6% vs. 2.9% 95% CI; 1.5–5.4%, p = 0.02). The frequency of malaria was found high in the age group between 2 and 14 years (10.3% 95% CI; 4.8–21.3%, p = 0.05) (Table 2).

**Table 2:** Socio- demographic characteristics and malaria among febrile study respondents (N = 444)

Factors	N tested	N ^+ve^ (%)	95% CI	p value
Gender				
Male	180	13 (7.2)	4.2–12.1	**0.01**
Female	264	6 (2.3)	1.0–5.0	
Age				
2–14	58	6 (10.3)	4.8–21.3	0.05
15–29	241	12 (4.0)	2.3–6.9	
30–44	111	0 (0.0)		
≥ 45	34	1 (2.9)	0.4–18.7	
Education				
Illiterate	201	7 (3.5)	1.7–7.1	0.45
Primary school and above	243	12 (4.9)	2.8–8.5	
Marital status				
Married	313	9 (2.9)	1.5–5.4	**0.02**
Non married	131	10 (7.6)	4.1–13.6	
Residence				
Urban	97	5 (5.2)	2.1–11.9	0 0.63
Rural	347	14 (4.0)	2.4–6.7	
Occupation				
Pastoralist	215	8 (3.7)	1.8–7.3	0.57
Others	229	11 (4.8)	2.7–8.5	

### Potential risk factors for brucellosis

At univariate logistic regression analysis, drinking raw milk and boiled milk (COR = 28.65, 95%CI: 3.86—212.42, p = 0.001) **(**COR = 19.25, 95%CI: 2.58—143.33, p = 0.004) respectively, drinking milk from aborted animal (COR = 2.87, 95% CI: 1.49—5.54, p = 0.002) and touching aborted fetus/discharges without protective equipment (COR = 2.82, 95%CI: 1.16—6.86, p = 0.022), were significantly associated with the occurrence of human brucellosis among these febrile patients (Table 3).

**Table 3:** Univariate analyses of potential risk factors for brucellosis of the study patients (N = 444)

Factors	N tested	Total + ve N (%)	COR (95% CI)	P value
Ownership of large ruminant				
No	333	49 (14.7)	1	
Yes	111	21 (18.9)	1.35 (0.77;2.37)	0.294
Ownership of small ruminant				
No	164	19 (11.6)	1	
Yes	280	51 (18.2)	1.70 (0.96;2.99)	0.066
Ownership of camel				
No	214	29 (13.6)	1	
Yes	230	41 (17.8)	1.38 (0.83;2.32)	0.218
Milk from large ruminant				
No	332	49 (14.7)	1	
Yes	112	21 (18.7)	0.78 (0.42;1.42)	0.419
Milk from small ruminant				
No	168	18 (10.7)	1	
Yes	276	52 (18.8)	1.12 (0.57;2.23)	0.725
Milk from camel				
No	214	29 (13.5)	1	
Yes	230	41 (17.8)	0.64 (0.35;1.17)	0.144
Type of milk use to drink				
None	96	1 (1.0)	1	
Raw	164	38 (23.2)	**28.65 (3.86;212.42)**	**0.001**
Boil	184	31 (16.9)	**19.25 (2.58;143.33)**	**0.004**
Drinking milk from aborted animal				
No	393	54 (13.7)	1	
Yes	51	16 (31.3)	**2.87 (1.49; 5.54)**	**0.002**
Touching of aborted materials/fetus				
No	391	51 (13.0)	1	
Yes	53	19 (35.8)	**2.82 (1.16;6.86)**	**0.022**

*COR* Crude odds ratio, *CI* confident interval

A multivariate logistic regression analysis model was built to measure the relationship between seropositivity for brucellosis and independent variables. All sociodemographic factors and potential risk factors that showed p-values < 0.05 in the univariate analysis were considered in the final multivariable logistic regression model. Being male (AOR = 2.41, 95%CI: 1.36**–**4.26, p < 0.002), drinking raw milk (AOR = 26.68, 95%CI: 3.22- 221.13, p = 0.002), boiled milk (AOR = 17.52, 95%CI: 2.06–149.04, p = 0.009) and touching aborted fetus/discharges without protective (AOR = 3.70, 95%CI: 1.61–8.50, p = 0.02) were associated with higher odds of having brucellosis infection among febrile patients (Table 4).

**Table 4:** Multivariable analysis of risk factors for occurrence of brucellosis of the study patients

Factors	Adjusted OR (95% CI)	P value
Gender		
Female	1	
Male	**2.41 (1.36;4.26)**	**0.002**
Education		
Illiterate	1	
Primary school and above	0.76 (0.40; 1.44)	0.399
Occupation		
Pastoralist	1	
Others	0.91 (0.45; 1.86)	0.779
Residence		
Urban	1	
Rural	1.19 (.53;2.64)	0.678
Type of animal owned		
None	1	
Large ruminant	1.27 (0.56;4.58)	0.710
Small ruminant	0.92 (0.28;2.97)	0.888
Camel	0.41 (0.09;1.78)	0.233
Two or more types of animals	0.49 (0.17;1.43)	0.192
Drinking milk from aborted animal		
No	1	
Yes	0.76 (0.28;2.02)	0.577
Touching of aborted materials/fetus		
No	**1**	
Yes	**2.56 (1.01;6.52)**	**0.048**
Type of milk used to drink		
None	**1**	
Raw	**26.68 (3.22; 221.13)**	**0.002**
Boil	**17.52 (2.06; 149.04)**	**0.009**

## Discussion

This institution-based cross-sectional study identified 140/444 (31.5%) positive by screening test (RBPT) for brucellosis, of which 70/140 (50%) of them were confirmed positive by ELISA. Among the fever patients in this study, the overall combined seroprevalence rate of brucellosis was 15.8% (95% CI; 12.7–19.7%), and the prevalence of malaria was 4.3% (95% CI; 2.7–6.6%). This study shows that in this malaria-endemic area, the prevalence of brucellosis among people with fever is higher than that of malaria. The possible reason for this difference is that malaria control has produced effective reductions in malaria incidence. On the other hand, human brucellosis has not yet been considered an important public health disease; consequently, the area was affected by febrile brucellosis.

The prevalence of *P. falciparum* was 4.3% and *P. vivax* was not detected. The result is lower than the previous health-institution-based studies carried out before the full implementation of the intervention programs in Ethiopia such as in 2013 (51.5%) [21], in 2015 (17%) [22], and in 2016 (43.8%) [23]. This significant reduction of malaria prevalence may be the impact of scaling up of malaria intervention programs towards elimination introduced since 2016 in the country [24]. Malaria infection was found common among male and young children which is most likely due to the fact that as observed males traditionally move from home for a short or long time camping along with livestock for grazing and naïve immunity of young children for malaria parasites. Even if the prevalence of malaria was found relatively low due to the prevention and control measures employed by the country to eliminate it from the country [24], sustainable devotion to control and prevention needs to be enhanced by addressing all infection. Because there would be a possibility of resurge of malaria epidemic and this identified *P. falciparum* which is the most severe of malaria may impact the health of the community in this study area.

The prevalence of human brucellosis in this study area is in agreement with the findings from febrile individuals of different Sub-Saharan African countries like Tanzania (15.4%), Northern Uganda (18.7%), and Northeastern Kenya (13.7%) [25–27]. This result revealed that human brucellosis is a febrile illness and highly circulating among sub-Saharan African countries including Ethiopia. The result was higher than the 2016 Ethiopian domestic animal brucellosis estimate, 5.3% in goats, 2.9% in cattle and camel, and 2.7% in sheep, but it was concurrent with the human estimates of pastoral area (17.4%) and higher than the human estimates of sedentary area (3.1%) [33]. This finding showed that the source of human brucellosis is most likely animals which are infected and served as reservoirs in this study area. The confirmatory finding of this study was lower than health facility studies in Borena (34.9%) of South Ethiopia and Metema (29.4%) of North Ethiopia [28], but it is quite higher than many previous findings of health facility-based studies among febrile individuals in southwestern Ethiopia, 1- 3.6% [22, 29] and in central Ethiopia, 2.15% [30]. The possible explanation for the difference in the seroprevalence could be due to the difference in the sampling design schemes used, the number of samples, exposure to *Brucella* species, and the type of diagnostic tests used.

The study identified residential area and gender as important risk factors for human brucellosis. Rural residents and being male who lived in this area were about three and a half and five and half times more likely to be seropositive for brucellosis compared to urban residents and females, respectively. This finding is in agreement with other studies in Uganda and Egypt [25, 31], which might be due to male individuals having frequent contact with animals than females. Besides, the study was showed that pastoralists were nearly six times more seropositive than other occupations, and having large ruminants, small ruminants, and/or camels were about more than ten times more likely seropositive for brucellosis than those who do not have any animals.

This study also identified consumption of raw milk and contacts with aborted fetus/discharges without protective equipment to be associated with brucellosis, which is in line with another couple of study findings in Uganda [25, 32]. This finding is supported by a WHO report which revealed contact with infected materials such as aborted fetus, placenta, urine, manure, and the carcass has been reported to cause human brucellosis in 60–70% of cases [2]. The study was revealed that consumption of boiled milk was a risk factor for brucellosis. The possible reason may be, during boiling, the milk might not boil up to the standard to kill the bacteria. The traditional habits of consumption of unpasteurized milk and fresh cheese and contamination of animal discharge are particularly common among remote areas like in this study area which requires attention to create awareness on the possible risk of acquiring *brucella* and other zoonotic infections.

This study has a few limitations. First, since it was a purposive cross-sectional study, we recruited only febrile individuals that visited health facilities that left behind apparently healthy chronic patients, and during self-reporting, there would be recall bias by the participants for possible factors associated to the occurrence of brucellosis in humans that weaken the inference of the finding. The other limitation is the test being based on serological tests; the reported seroprevalence of brucellosis could be difficult to differentiate from the previous infection.

## Conclusion

The prevalence of human brucellosis in fever patients in this study area is higher than the prevalence of malaria. The consumption of raw milk and contact with animal discharge can cause a significant risk of *Brucella* infection. So, the brucellosis disease must be sought in the differential diagnosis, like ELISA test that can be used to differentiate from other febrile diseases like malaria. Study is recommended to address asymptomatic brucellosis and to determine the circulating *Brucella* species and drugs profile, as well as the similarities and differences of species between humans and animals.

## Data Availability

The datasets used and/or analysed during the current study available from the corresponding author on reasonable request.

## Abbreviations

AHRI: Armeaur Hansen Research Institute
AOR: Adjusted odds ratio
COR: Crude odds ratio
ELISA: Enzyme linked immunosorbent assay
RBPT: Rose bengal plate test
WHO: World Health Organization

## References

[1] Tumwine G, Matovu E, Kabasa JD, Owiny DO, Majalija S (2015). Human brucellosis: sero-prevalence and associated risk factors in agro-pastoral communities of Kiboga District, Central Uganda. BMC Public Health.

[2] Corbel MJ, Alton JJ, Banal M, Diaz R, Dranovskaia, BA, Elberg SS et al. Brucellosis in humans and animals. WHO Press. 2006. ISBN:9241547138

[3] Dean AS, Crump L, Greter H, Schelling E, Zinsstag J (2012). Global burden of human brucellosis: a systematic review of disease frequency. PLoS Negl Trop Dis.

[4] Majalija S, Luyombo P, Tumwine G (2018). Sero prevalence and associated risk factors of Brucellosis among Malaria negative febrile out patients in Wakiso district, Central Uganda. BMC Res Notes.

[5] Gemechu R. Brucellosis and Its Control through One-Health Approaches in Ethiopia. 2017. J Vet Med Res 4 (3): 1080. ISSN:2378–931X

[6] Njeru J, Wareth G, Melzer F, Henning K, Pletz M, Heller R (2016). Systematic review of brucellosis in Kenya: disease frequency in humans and animals and risk factors for human infection. BMC Public Health.

[7] Bouley AJ, Biggs HM, Stoddard RA, Morrissey AB, Bartlett JA, Afwamba IA (2012). Brucellosis among Hospitalized Febrile Patients in Northern Tanzania. The Am J Trop Med and Hyg.

[8] Buzgan T, Karahocagil MK, Irmak H, Baran AI, Karsen H, Evirgen O (2010). Clinical manifestations and complications in 1028 cases of brucellosis: a retrospective evaluation and review of the literature. Int J Infec Dis..

[9] Migisha R, Nyehangane D, Boum Y, Page AL, Zúñiga-Ripa A, Conde-Álvarez R (2018). Prevalence and risk factors of brucellosis among febrile patients attending a community hospital in south western Uganda. SciRepo.

[10] Kunda J, Fitzpatrick J, Kazwala R, French NP, Shirima G, MacMillan A (2007). Health-seeking behavior of human brucellosis cases in rural Tanzania. BMC Public Health.

[11] Adhanom T, Deressa W, Witten H, Getachew A, Seboxa T, Birhanie Y, Hailemariam D, Kloos H (2006). Malaria. The Epidemiology and Ecology of Health and Disease in Ethiopia.

[12] PMI. President’s Malaria Initiative: Ethiopia - Malaria Operational Plan FY 2019. 2018. Source: CDC, US DOS, USAID

[13] Gumi B, Firdessa R, Yamuah L, Sori T, Tolosa T, Aseffa A (2013). Seroprevalence of Brucellosis and Q-Fever in Southeast Ethiopian Pastoral Livestock. J Vet Sci Med Diagn.

[14] Tschopp R, Bekele S, Moti T, Young D, Aseffa A (2015). Brucellosis and bovine tuberculosis prevalence in livestock from pastoralist communities adjacent to Awash National Park Ethiopia. Prev Vet Med.

[15] Yohannes M, Degefu H, Tolosa T, Belihu K, Cutler R, Cutler S (2013). Brucellosis in Ethiopia. Afr J Microbiol Res.

[16] Pappas G, Papadimitriou P, Akritidis N, Christou L, Tsianos EV (2006). The new global map of human brucellosis. Lancet Infe Dis.

[17] Zerfu B, Medhin G, Mamo G, Getahun G, Tschopp R, Legesse M (2018). Community-based prevalence of typhoid fever, typhus, brucellosis and malaria among symptomatic individuals in Afar Region Ethiopia. PLoS Negl Trop Dis..

[18] Federal Ministry of Health. National Malaria Guidelines, 3^rd^ Edn. Addis Ababa, Ethiopia, 2012. www.moh.gov.et/documents/26765/28899/National%2BMalaria%2BGuidelines/

[19] Diaz R, Casanova A, Ariza J, Moriyon I (2011). The Rose Bengal Test in human brucellosis: a neglected test for the diagnosis of a neglected disease. PLoS Negl Trop Dis..

[20] Hasibi M, Jafari S, Mortazavi H, Asadollahi M, Esmaeeli DG (2013). Determination of the accuracy and optimal cut-off point for ELISA test in diagnosis of human brucellosis in Iran. Acta Med Iran.

[21] Tadesse H, Tadesse K (2013). The etiology of febrile illnesses among febrile patients attending Felegeselam Health Center, Northwest Ethiopia. Am J Biomed Life Sci.

[22] Feleke SM, Animut A, Belay M (2015). Prevalence of Malaria among Acute Febrile Patients Clinically Suspected of Having Malaria in the Zeway Health Center Ethiopia. Jpn J Infect Dis.

[23] Hailu T, Alemu M, Mulu W, Abera B (2018). Incidence of Plasmodium infections and determinant factors among febrile children in a district of Northwest Ethiopia; a cross- sectional study. Trop Dis Travel Med Vacci.

[24] Deribew A, Dejene T, Kebede B, Assefa GT, Melaku YA, Misganaw A (2017). Incidence, prevalence and mortality rates of malaria in Ethiopia from 1990 to 2015: analysis of the global burden of diseases. Malar J.

[25] Muloki HN, Erume J, Owiny DO, Kungu JM, Nakavuma J, Ogeng D (2018). Prevalence and risk factors for brucellosis in prolonged fever patients in post-conflict Northern Uganda. Afr Health Sci.

[26] Mukhtar F. Brucellosis in a high-risk occupational group: sero-prevalence and analysis of risk factors. J Pak Med Assoc. 2010; 60 (12):1031–4. ISBN: 0030–9982 21381558

[27] Chipwaza B, Mhamphi GG, Ngatunga SD, Selemani M, Amuri M, Mugasa JP, Gwakisa PS (2015). Prevalence of bacterial febrile illnesses in children in Kilosa district Tanzania. PLoSNegl Trop Dis..

[28] Regassa G, Mekonnen D, Yamuah L, Tilahun H, Guta T, Gebreyohanis A et al. Human Brucellosis in Traditional Pastoral Communities in Ethiopia. Int J Trop Med 2009; 4 (2): 59–64. ref.20. ISSN: 1816–3319

[29] Tolosa T, Regassa F, Belihu K, Tizazu G. Brucellosis among patients with fever of unknown origin in Jimma University Hospital, Southwestern Ethiopia. Eth J Heal Sci. 2007; 17 (1):59–63. ISBN:1029–1857

[30] Tibeso G, Ibrahim N, Tolosa T (2014). Sero-prevalence of bovine and human brucellosis in Adami Tulu Central Ethiopia. World Appl Sci J.

[31] Lobna MA, Khoudair MR, Osman SA (2014). Sero diagnosis of brucellosis by using simple and rapid field tests with emphasis on some possible risk factors in humans. Global Vet.

[32] Migisha R, Nyehangane D, Boum Y, Zúñiga-Ripa A, Conde-Álvarez R, Bagenda F (2018). Prevalence and risk factors of brucellosis among febrile patients attending a community hospital in south western Uganda. Sci Rep..

[33] Tadesse G (2016). Brucellosis seropositivity in animals and humans in ethiopia: a metaanalysis. PLoS Negl Trop Dis.

